# Impact of Host Plant Connectivity, Crop Border and Patch Size on Adult Colorado Potato Beetle Retention

**DOI:** 10.1371/journal.pone.0095717

**Published:** 2014-05-09

**Authors:** Gilles Boiteau, Charles Vincent, Tracy C. Leskey, Bruce G. Colpitts, Pamela MacKinley, Doo-Hyung Lee

**Affiliations:** 1 Agriculture and Agri-Food Canada, Potato Research Centre, Fredericton, New Brunswick, Canada; 2 Agriculture and Agri-Food Canada, Horticulture Research and Development Centre, Saint-Jean-Sur-Richelieu, Quebec, Canada; 3 USDA-ARS, Appalachian Fruit Research Station, Kearneysville, West Virginia, United States of America; 4 Department of Electrical and Computer Engineering, University of New Brunswick, Fredericton, New Brunswick, Canada; United States Department of Agriculture, Beltsville Agricultural Research Center, United States of America

## Abstract

Tagged Colorado potato beetles (CPB), *Leptinotarsa decemlineata* (Say), were released on potato plants, *Solanum tuberosum* L., and tracked using a portable harmonic radar system to determine the impact of host plant spatial distribution on the tendency of the pest to remain on the colonized host plant or patch. Results confirmed the long residency time on the host plant and showed that close connection of the plant to neighboring plants hastened dispersal between plants. Tracking walking CPB for over 6 h in small potato plots revealed that all types of mixed borders tested (potato/bare ground, potato/timothy and potato/woodland) acted as a strong barrier and retained beetles within the patch. In another experiment in potato patches surrounded by bare ground borders, tracked walking CPB displayed similar behaviour for up to four days. The distribution of turning angles in the CPB walking paths was not uniform and corresponded to beetles following the edge rows of potato patches in response to the crop border barrier or reversing their direction as they reached the end of a row and therefore a border. Patch size had no or little effect on beetle retention in the patch. The relative distribution of counts of tagged beetles detected among small (16 m^2^), medium (64 m^2^) and large size (256 m^2^) patches of potato four days after initial release remained similar to that of numbers released. Even though mixed crop borders were a strong barrier to walking CPB emigrating from potato patches, the departure rate of beetles over time was high. Results suggest that the effect of mixed borders is largely limited to dispersal by walking and does not apply to beetles leaving host patches by flight. The manipulation of crop borders and patch size seem to have limited potential for the management of CPB emigrating from potato fields.

## Introduction

In Canada, potato farms average 150 ha but with rarely more than one third planted with the main crop [1]. Common land types adjacent to potato fields include bare ground fields or roads, grassy fields (pasture, timothy, cereals, etc.) and woodland. Insect dispersal through these fragmented agricultural landscapes is complex. The residence time of an insect foraging on a host plant and within the host patch in such fragmented areas depends on a complex set of factors (e.g. host or patch quality, patch location, accumulated residency time, thermoregulation, predation, reproductive behavior, population density.etc.) that determine the optimal staying time (see Pyke [2] for review). The landscape features can facilitate or impede insect movement among host plant patches and ultimately influence population dynamics [3]. Despite the importance of inter-field movement by insect pests, the subject remains understudied and its integration into crop management has been slow [4], [5]. This study addressed the first step of inter- patch (field) movement: moving away from the host plant, crossing the crop border and moving out of the patch.

The project was carried out using the Colorado potato beetle (CPB), *Leptinotarsa decemlineata* (Say). The CPB is an oligophagous species which has become an agricultural pest throughout most of the world, feeding almost exclusively on cultivated and wild Solanaceae, and is a threat to remaining potato growing areas [6]. Because of its oligophagous nature, the potato beetle is essentially an insular species on Solanaceous host plants surrounded by a fragmented area of mostly less suitable habitats. This study quantified how readily adult beetles abandon the host plant and their host patch. We looked at how the level of connectivity between the host plants (spatial distribution of plants) affected the dispersal of the CPB within the patch as well as how patch size and different vegetation across the border might impact emigration (sensu Skórka [7]) from the crop patch.

The CPB is a very mobile insect pest spreading throughout the world [8], [9]. It is both an active walker and flyer. Although there is some understanding of its seasonal dispersal [6], [10], [11], [12] and some attention given to colonization [6], [13], [14], less attention has been given to its residency on host plants [15], [16] and emigration from host patches [17], [18], [19]. From research on the limited potential of (early planted potato) trap crops to intercept overwintered beetles dispersing in the spring, we know that the insects are only partially retained by the trap crop before spreading rapidly to the adjacent potato crop [20], [21]. As on most other insect/crop systems, the natural retention of insects on trap plants is not sufficient and must be supplemented by the application of insecticides directly to the trap crop or the use of physical control methods such as vacuuming [22]. This relative inability of potato trap crops to retain adult CPB is in contrast with the stationary behavior described by Bach [15] and Boiteau and MacKinley [16]. In the latter study, 76% of CPB released on plant models of different architectural complexity remained on the models (made of polyvinyl chloride pipe) for the 6 h observation period [16]. This is an unexpectedly long period of residency considering that the models provided no nutrition. One possible explanation for the discrepancy between studies can be found from the effect of crop borders on insect dispersal. It is not uncommon for the type of vegetation found on either side of the boundary created by the interface of the crop and surrounding vegetation to affect boundary crossing rate of insects [23], [24]. The role of these mixed interfaces, referred here as mixed crop borders, at determining the level of CPB retention within the patch (compared to potato/potato borders) is essentially unknown. We do not know either if mixed crop borders could be used to help retain the beetles in the colonized crop to reduce spread between fields. Some studies have suggested that vegetation could slow down colonization of potato fields [17], [19], but only Schmera et al. [18] has provided evidence that potato plants surrounded by wheat could reduce emigration and dispersal by CPB between patches. Knowledge of inter-field movements at landscape levels is necessary to the development of sound IPM strategies. Identifying the boundary crossing rate or permeability of edges across a range of edge types and of different sizes of patches is a fundamental step to understand how edges affect dispersal of agricultural insects so that pest movement can be manipulated as part of IPM. In general, decreased permeability results in reduced dispersal and an opportunity for local control [25]. In addition, low levels of retention are likely to have little impact on the efficacy of pest management methods such as trap crops. However, high levels of retention should have a large impact as suggested by a mathematical model developed by [22].

The retention of dispersing insects on a crop is largely a function of its mobility within the crop, the permeability of the border, the size of the host patch and the dispersal path of that insect [4]. The probability that an individual crosses an edge that it has encountered (edge permeability) is the starting point of dispersal throughout the farm landscapes [26]. In spite of its importance, there is a surprising lack of information on this point [7], [25], [27]. This paucity of information is partly due to the difficulty of gathering field data. The inherent ability of the CPB to disappear from sight in the crop or surrounding vegetation makes it difficult to visually track individual paths within a plot and across borders. New tracking methods such as portable harmonic radar are making it easier to collect the information required to measure border interactions [3]. A combination of visual tracking using color markings or elytral punctures and harmonic radar tracking of tagged beetles made effective tracking possible [28]. A sound understanding of the relationship between the insect pest and the structure of its habitat is essential to develop a sound strategy to manage their within and between field dispersal. The tolerance of each species for within field changes as well as larger scale farm scale changes in the composition and distribution of crops will determine the importance of habitat manipulation as a pest management strategy [10], [19], [28].

Using tagged or marked CPB and a harmonic radar, three field experiments were conducted to determine the impact of plant connectivity (Experiment 1), patch border (Experiment 2), and patch size (Experiment 3) on beetle retention. Additionally, we aimed to determine the permeability of three different mixed borders.

## Materials and Methods

### Insects

Insects were obtained from a laboratory colony maintained at the Potato Research Centre, Fredericton, NB, in 2010 and 2012 and from potato fields receiving no insecticide sprays in 2011. Colony beetles were maintained indoors in cages on potato, *Solanum tuberosum* L., (cv. Shepody) under a 16L: 8D photoperiod. The age of the beetles ranged between 14 and 28 days old in 2010, and 15 to 21 days old in 2012. Beetles were sexed according to Rivnay [29]. In 2010, equal number of males and females were used in each treatment on each date but females with abdomen distended by eggs were excluded. We have shown in an earlier publication [30] that weight affects significantly the ability of beetles to take flight and can at the limit ground them. The logistics of releasing sufficient numbers of marked or tagged beetles to retrieve quantifiable data on their dispersal were such that the inclusion of a small percentage of individuals (gravid females) with significantly heavier weights could have limited the ability of the tests to detect differences between treatments. In 2011 and 2012, the potential confounding effects of weight differences [30] and oviposition behavior [31] on the dispersal behavior of the released CPB led us to focus the study to the dispersal of male beetle. Also, preliminary tests had revealed a greater tendency for the males than the females to disperse within potato plots. This approach removed potential confounding factors and optimized the experimental design for the beetles expected to have the most mobility.

In 2011, adult CPB were collected from the field the afternoon before each release, individually tagged and marked with color. The elytra of the beetles were lightly sanded and Quick Dry Wite-Out (Bic) was applied as a base for color spots made using markers (Sharpie). In 2010 and 2012, beetles were also individually tagged but marked using elytral punctures. The colored markers remained long enough on the beetles for the daily periods of observation in 2011. The permanent marking provided by the method of Unruh and Chauvin [32] was more appropriate for tracking beetles over days and weeks in 2012. In 2010 and 2011 beetles were maintained in small cages (2 L plastic containers – 15.2 cm h and a 15.2 cm diameter) provided with compound potato leaves inserted in water picks overnight but starved for 1 h before release. In 2012, due to the large number of beetles required, the beetles were maintained in groups of 75 in larger insect cages (40×40×76 cm) on potato plants (cv. Kennebec) but starved 1 h before release.

### Tags and harmonic radar

The vertical dipole tags used with CPB were similar to those described in Colpitts and Boiteau [33]. They consisted of a custom made 2 mm proximal pole followed by a 1 mm loop and a 6 mm pole made of AWG #34 copper wire weighing 2.1 mg and was attached to the pronotum of adults using a drop of Krazy Glue [34]. The harmonic radar used to track the tagged beetles, custom built by one of us (BC), consisted of a 4 kW pulsed marine magnetron oscillator operating at 9.41 GHz, low pass filter, and antenna as the transmitter [33].

### Experiment 1 - Impact of plant connectivity on beetle retention (2010)

#### Experimental plots

Treatments provided four levels of plant connectivity: (L1) A single plant isolated from other vegetation by at least 5 m of bare ground; (L2) Three potato plants transplanted in line so that mid-crown terminal leaflets overlapped; (L3) Three potato plants transplanted in line so that mid-crown terminal leaflets were 5 cm apart from each other; and (L4) a central plant in the middle of a potato patch approximately 12×12 m with rows 1 m apart and plants at 40 cm spacing. All treatment plots were separated from each other or other vegetation by at least 5 m of bare ground. Plots were established in a field section (LAT 45.9197, LON -66.6066) on the grounds of the Potato Research Centre in 2010. Potato plants (cv. Russet Burbank) were planted on 18 May 2010. The plants were protected from defoliation by CPB by the application of the organic insecticide Entrust 80 W (a.i. spinosad) at a rate of 50 g/ha. Entrust was never applied on plants or plots where beetles were being monitored and tests were not initiated in plots that received an application of Entrust until a sufficient period of time had elapsed to avoid insecticide residues. In 2010, beetles were released 17 d after the 30 June application, 18 d after the 9 July application and 25–41 d after the 16 July application. Whenever plants were damaged or showed >10% defoliation (arbitrary threshold), releases and observations were carried out on duplicates of the initial treatment plots (established at the same time as the initial plots) to ensure that plant damage or defoliation did not affect the movement of the beetles.

#### Procedure

Tagged beetles were released individually on a stem in the middle of the canopy of the release potato plant in each treatment. Beetles were released with care to minimize stress to the insect. The operator let each CPB grasp a wooden rod used as support to move the insect from the container to a stem. Beetles were released on the middle plant of the connected and spaced groups, the isolated single plant and a central plant in the middle of the potato plot. One insect was released on each one of the four treatments at 3 min interval (random selection of treatments) and radar monitoring carried out every 12 min thereafter for 6 h or until the insects had walked or flown from the plant/set of plants/plot.

During each monitoring session, the plot and associated plant or plants were scanned visually and with the radar from all angles for up to 3 min to try and locate the tagged insect and determine if it had moved and if so where. The presence/absence of tagged released beetles was recorded on the single plant treatment (L1). In the connected (L2) and spaced (L3) treatments, plants were numbered and the location of the tagged beetles recorded throughout the monitoring. In the case of the central plant in the middle of a potato plot (L4), the location of the tagged beetle was recorded according to a numbered grid of the plants. In all cases, the scout recorded whether the beetle had been found on the plant or on the ground.

#### Data analysis

Residency was expressed as the proportion of tagged beetles remaining on a plant or treatment over a given period of time as the period of time that elapsed between the time of release and the time the tagged insect was observed to have moved away. In cases when residency extended beyond active tracking such as overnight, elapsed time was summed up over days. If the insect was at the same location as in the previous monitoring session, it was presumed to have remained at that location and the time elapsed added to the residency period. If the insect had moved to a new location, half of the elapsed time was assigned to the last recorded location.

The proportion of beetles remaining on the release plant throughout the day after release in each plant treatment was compared to an even distribution using a Chi-Square test. The mean residency time on each release plant and each treatment on the day of release were analysed using a one-way ANOVA followed by a Tukey HSD test when appropriate. The mean residency time over days for combined and separate sexes was analyzed using one-way ANOVA followed by Tukey HSD whenever appropriate. The residency time of males and females within each treatment was compared using Students t test. The analysis was carried out using VassarStats (www.faculty.vssar.edu/lowry/VassarStats.html) and [35].

### Experiment 2 - Impact of patch border on beetle retention (2011)

#### Experimental plots

**Figure 1:**
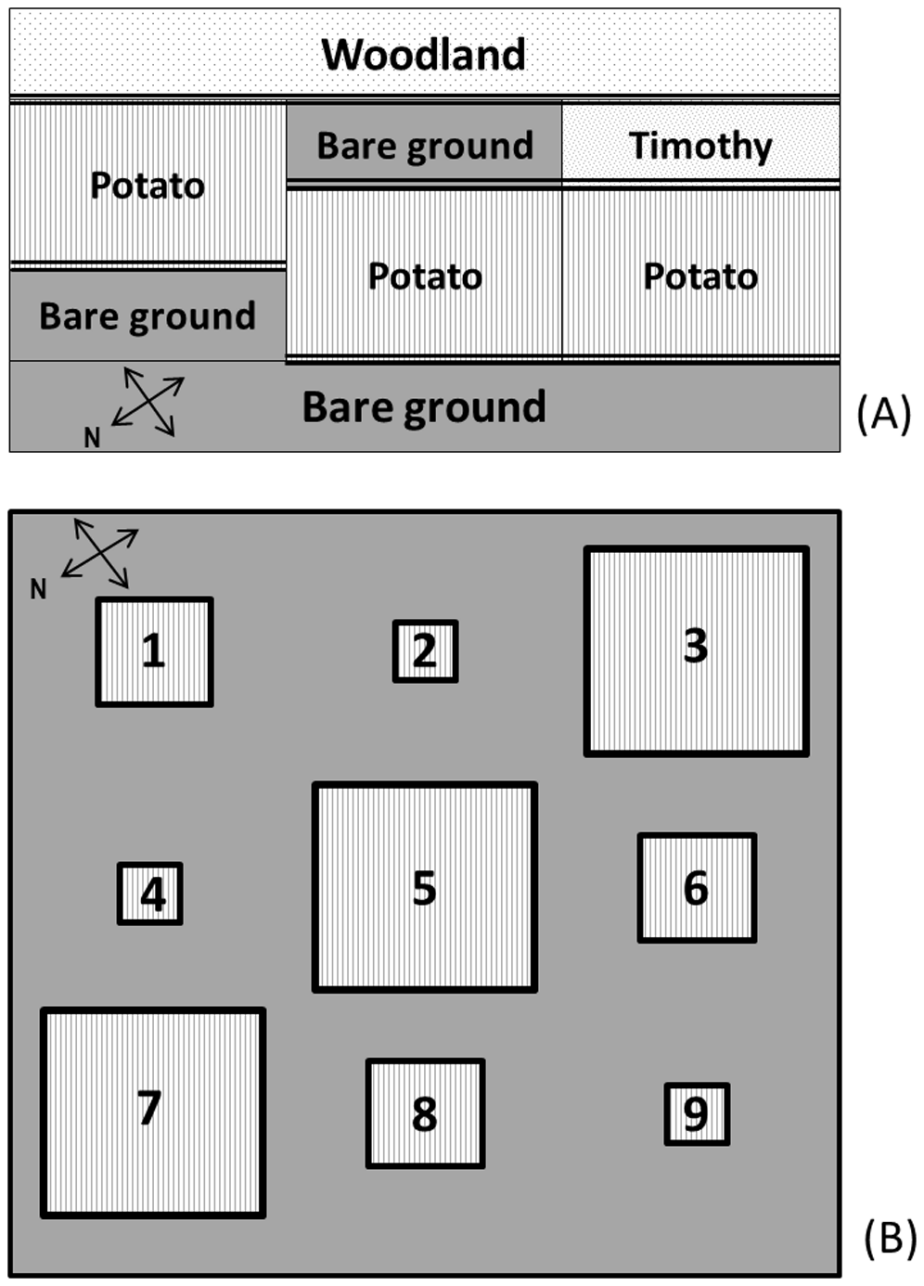
Diagrammatic representation of field plots in 2011 (A) and 2012 (B). Mixed borders are represented by  =  =  =  =  in (A). In 2012, plots 2, 4, 9 were 4×4 m, 1, 6, 8 were 8×8 m and 3, 5, 7 were 16×16 m (see also Table 1).

Two experimental sites (LAT 45.9134, LON -66.6076 and LAT 45.9183, LON -66.6029), each made up of three contiguous potato fields, approximately 16 m×40 m, were established on the grounds of the Potato Research Centre in 2011. One field was immediately adjacent, on one side, to woodland. The other two fields were separated from the woodland by a 5×40 m bare ground field and timothy field, respectively (Fig. 1A). Potato rows were perpendicular to the mixed border. Plots measuring 3×3 m were flagged in the potato crop with one side of the plot along the mixed border (adapted from Ries and Debinski [4]). The potato crop was planted as close as possible to the woodland, bare ground and timothy plots to enhance the contrast between the habitats. The mixed border treatments consisted of: potato/woodland, potato/timothy, potato/soil (bare ground) or potato/soil (bare ground) rotated 180° from the previous one. Plots were at least 8 m away from any field corner. Releases were carried out on different plots within each treatment to minimize the risk of plant damage affecting the movement of the beetles within the plots. The plants were protected from defoliation by CPB by the application of the organic insecticide Entrust 80 W (a.i. spinosad) at a rate of 50 g/ha. Entrust was never applied on plots where beetles were being monitored and tests were not initiated in plots that received an application of Entrust until a sufficient period of time had elapsed to avoid insecticide residues. In 2011, only half of the potato area at each site was sprayed with Entrust and beetles were released a minimum of 20 d after the 8 July and 15 July applications.

#### Procedure

Groups of 12 to 20 beetles were released in late afternoon the day before observations were to be carried out on a potato plant in the middle of a plot and a cage (57×57×60 cm) placed on top of the plant. Preliminary tests had revealed a tendency for beetles to remain on the release plant for some time before engaging into dispersal. The early release allowed the beetles to adapt to the environment. The observations started in mid-morning with the removal of the cage. The path of each beetle monitored, visually and with the portable harmonic radar, was recorded on a scaled map showing the location of each plant and borders. Tracking was continuous to prevent loss of beetles and because crossing and/or return could take place over a short period of time and be overlooked. The number of beetles crossing, approaching or avoiding the boundary were recorded at each border for each treatment. The number of beetles crossing back was also recorded. For logistical reasons, all observations were carried out between 10:00 and 17:00 h on the first site between 27 June and 23 August 2011 and on the second site between 24 and 26 August 2011, except on rainy days.

The small size of the plot and the number of beetles released maximized encounters between beetles and borders within the daily period of observation. Responses of walking beetles at the mixed border were compared to responses at the three potato/potato borders of the plot. The border was considered crossed whenever the tagged beetle walked across the mixed border into the woodland, timothy or bare ground plot itself. The canopy line represented the perimeter of woodland, timothy and fields. The width of the imaginary lines separating each potato plot from the rest of the potato field was half the width of the canopy of the potato plants, within and between rows. Because behavior at the borders was the focus of our study, beetles that did not disperse from the release plant were not considered in the analysis.

Tracking of each beetle continued if encounters with potato or mixed borders that did not result in a crossing. However, when a beetle crossed a mixed border or potato crop border by a distance of at least 1 m or 1 h had elapsed, tracking was discontinued.

It was logistically impossible to monitor and record the dispersal of beetles on more than one plot (and therefore one border treatment) at a time on any given day. The order of observations was chosen randomly over time (days) and repeated throughout the crop season.

#### Data analysis

Beetle responses at each mixed border were compared to responses at potato/potato borders (i.e. control) of the plots [4]. Dispersal paths for each beetle were graphed and examined to determine if beetles that did not cross a border showed any indication of having approached a border or approached and avoided a border. Expectations of random frequencies for crossings were based on the fact that each edge constituted 25% of the total perimeter of the plot. A chi-square test was used to determine whether individuals crossed mixed borders more frequently than the control borders (potato/potato) of the plot.

### Experiment 3 - Impact of patch size on beetle retention (2012)

#### Experimental plots

Plots measuring 4×4 m, 8×8 m and 16×16 m were set up in three blocks of three plots in each of two fields (LAT 45.9230, LON -66.6135 and LAT 45.9197, LON -66.6066) on the grounds of the Potato Research Centre in 2012 (Fig. 1B; Table 1). Plots within a block were completely randomized and replicated across weeks throughout the summer. Plots were set in two fields. There were nine replicates: the first three replicates and the last three replicates were carried out in one field and the middle three replicates in a separate field. The plants were protected from defoliation by CPB by the application of the organic insecticide Entrust 80 W (a.i. spinosad) at a rate of 50 g/ha. Entrust was never applied on plots where beetles were being monitored and tests were not initiated in plots that received an application of Entrust until a sufficient period of time had elapsed to avoid insecticide residues. In 2012, beetles were released 12 d after the 29 June application, 11d after the 13 July application and 11–31 d after the 20 July application. Patches were immediately surrounded by 1 m (middle of potato hill to edge of trench was 1 m) of bare ground followed by a plastic lined trench [36] used to trap tagged beetles walking in or out of the plots.

**Table 1:** Characteristics of potato plots used for the release of electronically tagged and marked Colorado potato beetles in 2012.

Treatment Plot	Size (m)	Surface (m^2^)	Crop (row-m)	Perimeter (m)	Releases^1^
Small	4×4	16	14	16	5
Medium	8×8	64	56	32	20
Large	16×16	256	226	64	80

1 Number of tagged Colorado potato beetles released daily over three consecutive days each test week.

#### Procedure

Tagged and marked beetles were released daily at a density of 1.2 adult CPB for 10 plants over three consecutive days for a total of 3.6 adult CPB per week for 10 plants. Releases were spread out over three days for logistical reasons (assembly of tags). Groups of beetles were released according to treatments early on the morning (9:00–10:00) on a plant in the middle of each plot and a cage placed on top of the plant. Tagged beetles were released close to a border to encourage encounters between beetles and borders. They were released in the center row of each plot, four plants in (from the end of that row) except for the first week when they were released in the center of the plots. The observations started in mid-morning with the removal of the cages. The observer moved randomly between the three plots locating the new position of selected beetles visually and with a portable harmonic radar. Up to three beetles actively dispersing were selected in each plot and their position recorded on a scaled map showing the location of each plant and borders. Tracking continued for 15 min in each plot continuously throughout the day (10:00 to 16:00). The procedure was repeated on Day 2 and Day 3. Between the installation of the release cages and 10:00 as well as at 16:00, the trenches were monitored for tagged/marked beetles and the crop rows radar monitored to estimate the number of remaining tagged beetles and their position. On Day 4 and Day 5, there were no releases but trench and field monitoring as well as path tracking continued.

Retention or residency was measured in the morning of the day following each release (Day 2 – residency of 1 day; Day 3 – residency of up to 2 days; Day 4 – residency of up to 3 days) and at the end of day 5 (Day 5 – residency of up to 4 days). In cases when residency extended beyond active tracking such as overnight, retention was accumulated over days. If the insect was detected in the same plot after failed detections, it was presumed to have remained at that location and the time added to the retention period.

#### Data analysis

The number of beetles walking off (trench counts), the number of resident tagged CPB (radar monitoring) and the walking pattern were related to the size of the plots.

Observations in 2011 were limited to small size plots and releases into a single plot per day to allow for tracking of the tagged beetles without interruptions. In 2012, it was impossible to track individual beetles without interruptions because of the larger number of plots, the addition of medium and large size plots and the extension of the observation period to up to 5 d after release. Individuals were tracked as continuously as possible. However, it was necessary to assume that tagged beetles moved in a linear direction between any two consecutive 30–45 min detection intervals as well as overnight when putting together walking tracks.

The impact of the border on the walking track of the beetles was analyzed by classifying all moves as either toward or away from the border for each patch size. The straight-line distance between the start and endpoints of each path was measured and mean displacement for each plot size were calculated and compared by ANOVA.

The frequency distribution of turning angles shown over the range of −180° to +180° in figures in the text was transcribed in six categories of 30° from 0° to +180°. Because turning angles and absolute directions are circular quantities, −180° and +180° are considered the same [37]. The distributions in small, medium, and large plots were compared to a uniform distribution as well as among themselves using a chi square test. It was physically impossible to monitor the occasional and quick CPB flight departures for systematic comparison with the relatively long bouts of walking dispersal. The departure rate of CPB from the plots that could not be accounted for from observations of the plants and plastic lined trenches was assumed to have taken place by flight.

## Results

### Experiment 1 - Impact of plant connectivity on beetle retention (2010)

#### Residency on release day

Beetles that left their treatment on day 1 in the course of the first 6 h of observation had average residency periods of 150 to 347 min (Table 2). The residency time was significantly longer (F_3, 50_ = 6.84; P = 0.00059) on treatments with a high level of connectivity (L2 and L3) than on single plants isolated (L1) or in a plot (L4). The residency time on the release plant itself was not statistically different (F_3, 50_ = 1.52; P = 0.221) across treatments (Table 2).

**Table 2:** Mean residency time (min ± S.E.) of tagged adult Colorado potato beetles on potato plants with different levels of connectivity.

	Residency time on release day		Residency time across days					
	Release plant	All treatment plants	All treatment plants					
Connectivity levels (L)	♂ and ♀ Mean^1^ (min ± SE)	♂ and ♀ Mean^1^ (min ± SE)	♂ and ♀ Mean^1^ (min ± SE)	♂ Mean^1^^,^ 2 (min ± SE)	♀ Mean^1^^,^ 2 (min ± SE)	df	t	P
L1 - 1 plant isolated	150±36a	150±36a	2246±524a	1326±394aB	3308±972aA	16	1.887	0.039
L2 - 3 plants connected	194±46a	322±55b	1550±337a	844±402aB	2412±434aA	17	2.658	0.017
L3 - 3 plants spaced	253±41a	347±55b	1558±386a	1074±334aA	2163±784aA	15	1.332	0.203
L4 - 1 plant in plot	150±21a	150±21a	893±251a	307±119aB	1479±441aA	15	2.565	0.021

Beetles that remained on their respective treatment plants overnight were not included in the calculation of residency time on release day.

1 Means within a column followed by different letters are significantly different at P  =  0.05.

2 Means for ♂ and ♀ within the same row followed by different capital letters are significantly different at P  =  0.05 according to Student's t test (shown in nearby columns).

Although L2 and L3 had a similar high residency time, the proportion of beetles remaining on the release plant in L2 (3 connected plants) was less than half that remaining on the release plant in L3 (3 spaced plants) at the end of 6 h of observation (on day 1). L2 had the lowest proportion of beetles remaining on the release plant at the end of 6 h of observation (on day 1) (χ^2^ = 15.66, df = 3, P = 0.0013). Twenty-four percent (5/22) of the beetles on the three connected plants treatment (L2) remained on the release plant compared to 58% (15/26) on the three spaced plants (L3) and 67% (19/28) on the single plant (L1 - control). In the potato plot treatment (L4), where plant canopy progressed from spaced to overlapping over the season as plants grew, the number of beetles remaining on the release plant was intermediate at 39%.

For beetles released on the isolated one plant treatment (L1) and the potato plot treatment (L4), none of the representative 28 individuals moved back after leaving them. There was, however, considerable back and forth movement between the release plant and the two side plants making up the three connected plant (L2) and the three spaced plant (L3) treatments. In the three connected plant treatment (L2), between the release and the end of the day or before leaving the treatment, 16 of the beetles relocated 69 times between the three plants: 6 and 32 times to the side plants, and 31 times to the middle (release) plant. In the three spaced plants treatment (L3), 11 of the 27 beetles relocated 43 times between the three plants: 6 and 8 times to the side plants and 29 times to the middle plant.

#### Residency across days

Assuming that beetles that stayed overnight remained on the same treatment plants until observations resumed the next day, the mean total residency time for beetles (sexes combined) on a treatment was highly variable and did not differ statistically between treatments (F _3, 99_ = 2.1; P = 0.105) (Table 2). Some dispersal could have taken place in the evening and early morning hours if the temperature was suitable but not at night [8]. The mean total residency times on each treatment did not differ either when males and females were considered separately (Males: F _3, 51_ = 1.82; P = 0.155; Females: F _3, 44_ = 1.24; P = 0.307). However, the mean total residency time of females was significantly longer than that of males in three of the four treatments (Table 2).

The mean total residency time was not related to change in the size of plants (Height: 12–62 cm) throughout the season (one plant: R^2^ = 0.059, F_1, 26_ = 1.631, P = 0.213; three spaced plants: R^2^ = 0.034, F_1, 25_ = 0.883, P = 0.356; three connected plants: R^2^ = 0.066, F_1, 18_ = 1.276; P = 0.273; Potato plot: R^2^ = 0.0087, F_1, 26_ = 0.227, P = 0.638).

### Experiment 2 - Impact of patch border on beetle retention (2011)

**Figure 2:**
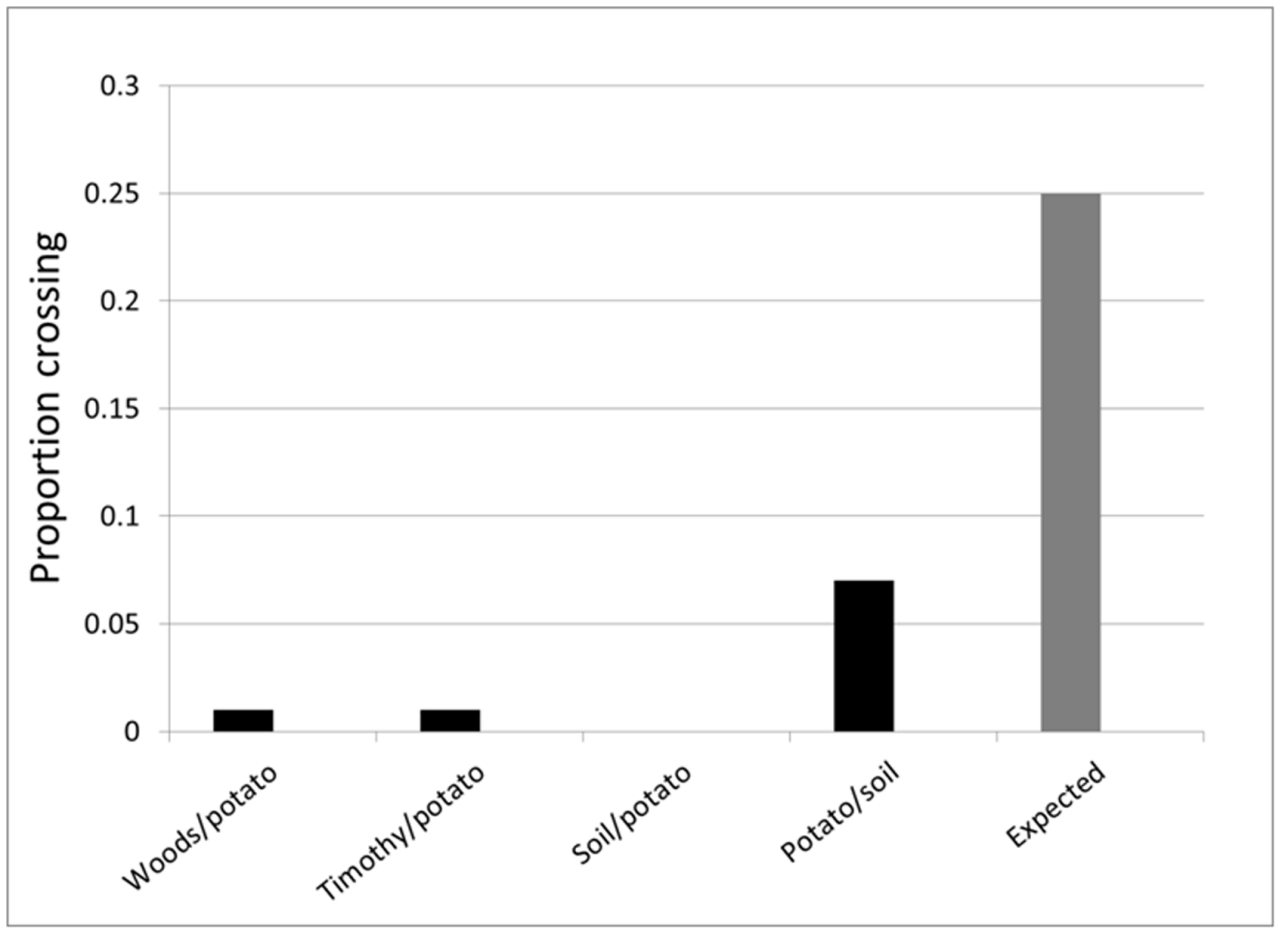
Proportion of Colorado potato beetle crossing a mixed border. Proportion of *Leptinotarsa decemlineata* leaving the potato plot by crossing the mixed border: woodland, timothy, soil (bare ground) or soil (bare ground) rotated 180° from the previous one. Values <0.25 indicate a bias against crossing the border because the mixed border constitutes 25% of the perimeter of the potato plot. Crossings at the potato/soil border did not differ significantly from random (χ^2^ = 9.56, df = 1, P = 0.002). Differences between expected and observed values for the other treatments could not be tested using a chi-square goodness-of-fit test because of the low crossing frequencies in spite of the large number of replications.

Data revealed a strong barrier effect of mixed borders. Only eight of the 341 beetles (2%) tracked until they had left the potato plot crossed through the mixed borders; all others dispersed within the adjacent potato field (Fig. 2). The response was similar across the four mixed borders tested: potato/woodland, potato/timothy, potato/bare ground and potato/bare ground (180°). The slightly higher frequency of crossings in the potato/bare ground (180°) mixed border treatment (14) furthest from the woodland than in the potato/bare ground mixed border treatment (0) closest to the woodland suggest that the response of the beetles to mixed borders could have been affected by the 180° difference in the orientation of the plots. However, regardless of whether the increased number of crossings reflected attempts by beetles to avoid the silhouette of the woodland [38], [39] or to orient in the direction of the sun [8], [40], the overall frequency of crossings of the potato/bare ground border remained below that expected from a random distribution of crossings, confirming the impermeability of the bare ground edge. The number of back crossings at potato/potato borders along the interior of the potato plot was low (17/333) averaging 0.05±0.006 across treatments. No back crossings were recorded at mixed borders in part due to the very low number (8) of mixed border crossings.

The barrier effect of the crop boundaries can be expressed in terms of edge permeability index defined by Stamps [26] as the proportion of individuals approaching an edge that cross it. In our case, interior potato - potato borders had a high permeability index (0.95) but mixed borders had low permeability indices: woodland, 0.01; timothy, 0.01; bare ground, 0.00; bare ground (180°), 0.17. Permeability indices were similar to the frequency of crossings because the crop border was highly permeable whereas the other types of border tested were all highly impermeable.

### Experiment 3 - Impact of patch size on beetle retention (2012)

The size of the host patch did not influence the proportion of tagged CPB retained in the release patch. The distribution between small, medium and large size patches of potato of the total numbers of these tagged beetles detected by radar 1, 2, 3 or 4 days after initial release remained similar to the 1∶4∶16 distribution ratio used in all beetle releases of June - July and August (Table 3). There was also no change in distribution for July - August except on Day 4 with almost twice as many tagged beetles than expected in the small (Table 3).

**Table 3:** Distribution between small, medium and large size patches of potato of the total numbers of tagged beetles detected by radar 1, 2, 3 or 4 days after initial release in three consecutive replicates.

	Total # detected			
Treatment (patch size)	Day 1	Day 2	Day 3	Day 4
Replicate 1 (June-July)	Release 1	Release 2	Release 3	No release
Small	3	10	21	17
Medium	27	59	69	47
Large	102	193	257	271
Total	132	262	347	335
χ^2^	1.88	3.26	1.26	5.42
df	2	2	2	2
P	0.391	0.196	0.533	0.0665
**Replicate 2 (July-August)**				
Small	6	9	6	17
Medium	39	29	36	33
Large	128	142	151	129
Total	173	180	193	179
χ^2^	2.22	1.72	1.06	7.63
df	2	2	2	2
P	0.33	0.423	0.589	0.022
**Replicate 3 (August)**				
Small	4	8	13	16
Medium	29	51	56	61
Large	97	186	203	206
Total	130	246	272	283
χ^2^	1.8	1.53	1.62	2.37
df	2	2	2	2
P	0.406	0.465	0.445	0.306
**Replicates combined**				
Small	13	27	40	50
Medium	95	139	161	141
Large	327	522	611	606
Total	435	688	812	797
χ^2^	5.45	1.46	1.04	4.59
df	2	2	2	2
P	0.066	0.482	0.575	0.101

The distribution of the detection numbers on any given day was compared with that of the released beetles (1:4:16 for small: medium: large plots)^1^ using a Chi-Square test.

1 A total of 45, 180 and 720 adult Colorado potato beetles were released each day (except day 4) on small, medium and large plots respectively (Table 1) for a total of 135, 540 and 2160 over the three replicates.

**Figure 3:**
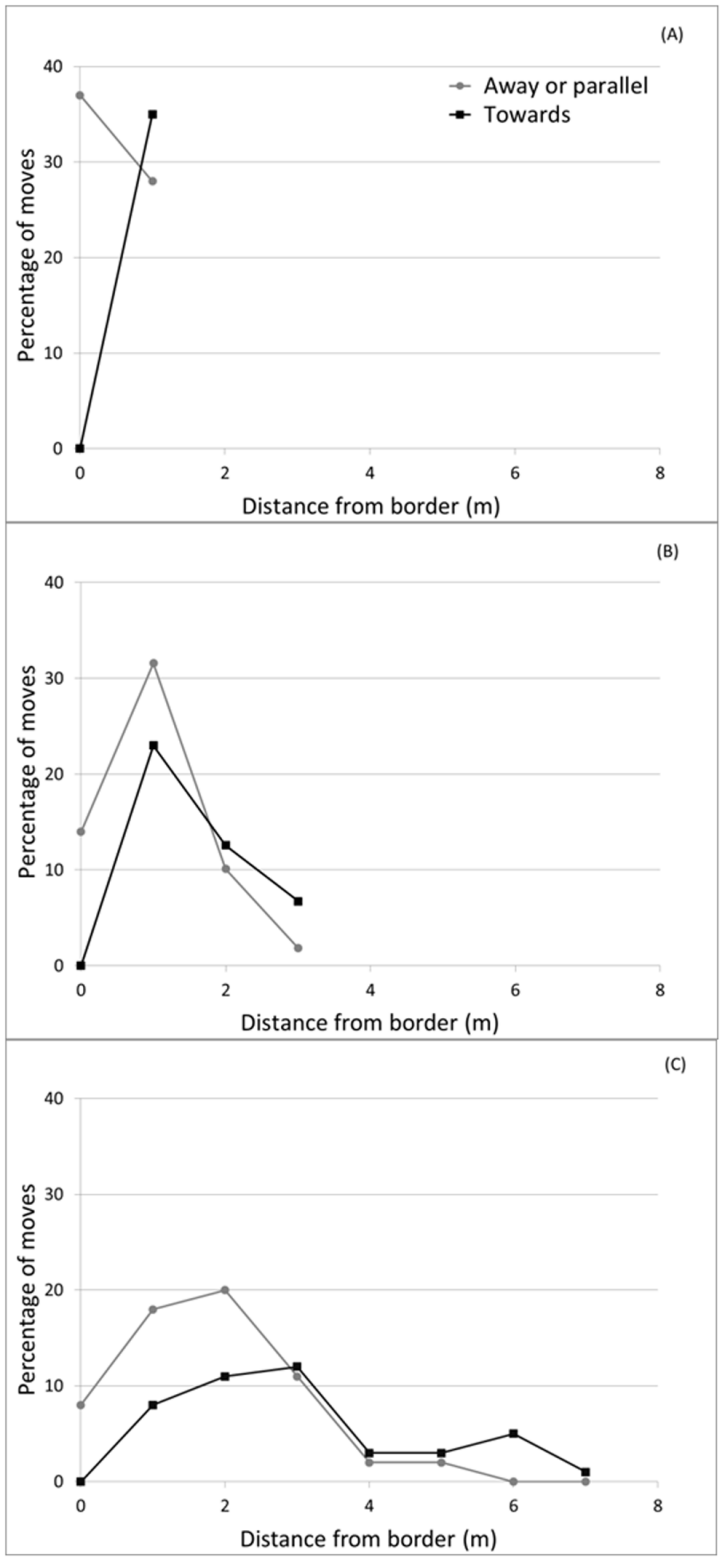
Walking moves away and towards potato/bare ground borders. Percentage of total moves by tracked adult Colorado potato beetles oriented away and towards bare ground borders in small (A), medium (B) and large (C) size potato plots (as described in Table 1) in 2012. The border row starts at 0 m.

**Figure 4:**
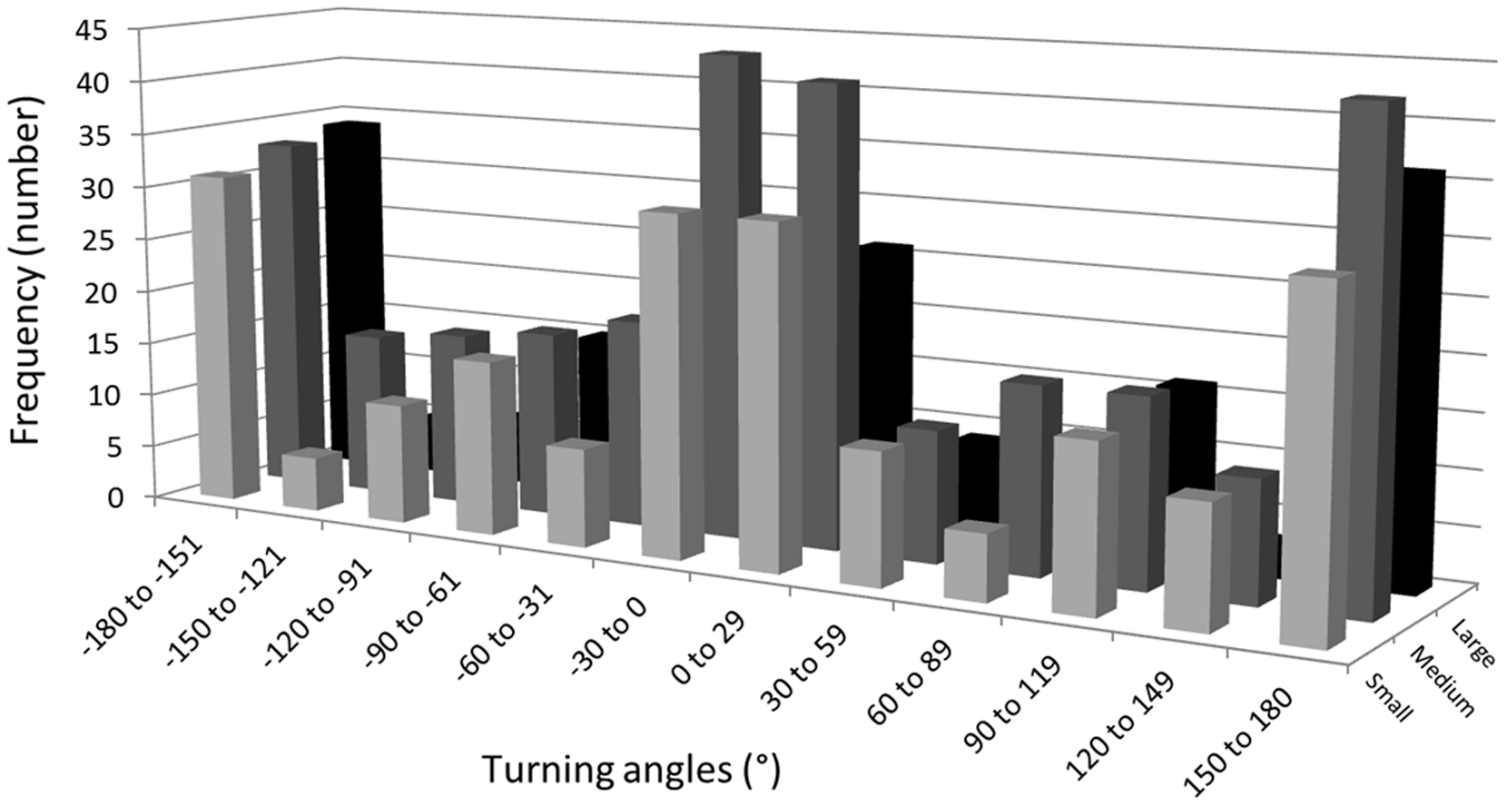
Distribution of turning angles for walking Colorado potato beetles. The frequency (number) distribution of turning angles (degrees) characterizing the movement of tagged adult Colorado potato beetles (pooled data) in small, medium, and large potato patches throughout the summer of 2012 in Fredericton.

The movement pattern of tracked walking CPB within these plots was consistent with the results of the 2011 experiment where the mixed border acted as a barrier to the walking dispersal of the beetles out of the plots. The mean displacement (shortest distance from release to final point) of tagged adult CPB tracked with the radar increased significantly (F_2, 222_ = 16.11, P<0.0001) with the size of the plot: small, 1.83±0.12, N = 60; medium, 2.78±0.20, N = 71; large, 3.75±0.28 meters; N = 92. The exploration (displacement) of the host patch by the beetles over the five day period was proportional to the size of the plot (Y = 124.87; X – 233.5; r^2^ = 0.87). Between 58 and 65% of total moves were directed away from the borders and this was independent of plot size (Table 4). The proportion of moves directed away from the border or parallel to the border increased sharply as the distance from the border became smaller (Fig. 3) regardless of plot size. None of the tracked beetles that had reached a border row or an end of row plant were observed crossing over to the bare ground surrounding the plots. The proportion of total moves peaked away from crop edges and the proportion of moves away from edges was consistently larger than that of moves towards edges for beetles located at crop edge (Fig. 3). The frequency distribution of turning angles differed from that for a uniform distribution in small (χ^2^ = 63.7, df = 5, P<0.0001), medium (χ^2^ = 71.7, df = 5, P<0.0001) and large (χ^2^ = 90.4, df = 5, P<0.0001) plots (Fig. 4). However, the frequency distribution of turning angles in medium plots and large plots did not differ significantly from that in small plots (χ^2^ = 2.4, df = 5, P = 0.788 and χ^2^ = 9.6, df = 5, P = 0.088, respectively). The movement of beetles was characterized by a degree of linearity in successive moves as indicated by the distribution of turning angles. The high frequency of turning angles centered on zero shows that beetles tended to move more or less in one direction and not a random path which would be characterized by a more or less uniform distribution of turning angles. It would correspond to beetles following the edge rows of potato plots in response to the crop border barrier. In addition, it is not uncommon in row crops for walking insects to follow rows. The almost equally high frequency of turning angles at −180° and +180° observed here corresponded to beetles reversing their direction as they reached the end of a row, and therefore a border.

**Table 4:** Influence of patch size on the movement pattern of walking adult Colorado potato beetles in relation to the crop borders.

	Potato plots		
	Small	Medium	Large
Walking tracks (#)	27	41	30
Moves (total #)	235	326	188
Moves away from border	152	188	116
Moves toward border	83	138	72
Moves away/total moves (%)	65	58	62
χ^2^ for large plot ratio (away/toward)	2.57	0.04	NA
df	1	1	NA
P	0.109	0.842	NA

The ratio of the number of moves away from borders of small and medium patches to moves toward borders of small and medium patches by tagged beetles tracked by harmonic radar during 4 days after release throughout the summer of 2012 was tested against the same ratio in large patches using a Chi Square test.

The negative impact of mixed borders on emigration of walking beetles was further supported by the recovery of only 74 tagged beetles from the trenches surrounding each plot over the 20 days following each release (2.0% of beetles released). Fifty-seven of the 74 beetles were captured after walking out of the corresponding plot. Seventeen originated from other plots and had therefore likely flown out of the plots they had been released in and were caught as they walked to colonize other plots. As expected, the number of walking emigrating beetles retrieved from the trenches surrounding their plots was proportional to plot size (perimeter) with 10, 19 and 28 CPB from small, medium and large plots, respectively (χ^2^ = 0.99, df = 2, P = 0.610).

However, in spite of all the evidence for a high number of walking beetles having been directed back into the potato patches as they encountered mixed-crop borders approximately ⅔ of beetles released ended up moving out of the plots during the experiment. Only 28% of the 2835 tagged CPB released throughout summer 2012 remained in their initial potato patches by the end of the experiment (46% (435/945) on Day 1; 36% (688/1890) on Day 2; 29% (812/2835) on Day 3 and 28% (797/2835) on Day 4). Considering the evidence for borders acting as barriers to CPB emigration by walking, it is likely that the high level of departure occurred by flight rather than by walking.

## Discussion

### Host plant residency, mixed borders and host patch size

The level of insect pest retention by the host plant can significantly affect population dynamics, spread in the crop and in the landscape, economic yield loss and the effectiveness of control strategies [2], [3], [41], [42]. In the case of the CPB, there is evidence that adults tend to remain on a potato plant for some time after its colonization before dispersing to other potato plants [15], [16]. The trends reported in Bach [15] emphasize the relative lack of movement in the adult CPB compared to other specialist chrysomelids such as the striped cucumber beetle, *Acalymma cittata* (Fab.). On plant models, this stationary behavior of the CPB translated in a strong majority of released beetles spending more than 6h on the same model [16]. Results of our 2010 field experiment confirmed the stationary behavior but also demonstrated an impact of host plant connectivity on CPB daily retention. Two thirds of the beetles remained on single isolated release plants for 6 h or more (L1) compared to one quarter on release plants connected to two lateral plants (L2). In the single plant treatment, the outer canopy of the plant seemed to act as a boundary as the beetle explored the plant and the absence of other nearby plants when the beetle descended back on the soil at the base of the plant led to it climbing back on the stem. In the connected treatment (L2), the leaf bridges to the lateral plants broke the outer canopy boundary and the nearby two plants were readily climbed on when the beetle descended back on the soil at the base of the release plant. In the spaced plant treatment (L3), the outer canopy of the release plant seemed to act as a boundary as in the single plant treatment but the lateral plant were close enough to “catch” some of the beetles that descended back on the soil at the base of the release plant. The slightly more than one third of the beetles remaining on the release plant in the potato plot treatment probably resulted from plants changing from isolated to spaced and then connected as the plants grew throughout the season.

Of the beetles that moved away from the treatments on the first day, those on the single plant (L1) did so the earliest but those on the three connected (L2) and three spaced plant treatments (L3) only did so after twice as much time having the opportunity to explore neighbouring plants. It is not clear why beetles moving away from the plant in the potato plot (L4) did so as fast as the single plant treatment but it suggests that small differences in inter-plant distance have a strong impact on the level of dispersal. It is possible that beetles perceived no edge or boundary and therefore were more willing to forage more freely and quickly. Results demonstrate that plant connectivity is responsible for potato beetles moving away from the release point within the release row rather than across rows in all releases as noted by Szendrei [19]. However, the absence of a difference between treatments in mean residency time across days indicates that the impact of plant connectivity on inter-plant movement can be largely restricted to a short period of time (e.g., release day). The presence of lateral plants, only accessible from the ground slightly decreased the proportion retained on the release plant and resulted in relocation to lateral plant. The presence of lateral plants touching the release plants decreased the proportion of beetles remaining on the middle plant by more than half and substantially increased the proportion on lateral plants. Essentially, results suggest that more beetles will tend to remain on the release plant for the first 6–10 h if it is isolated. Presuming that the release of a beetle on a plant is similar to colonization, it would seem that in the absence of visual or olfactory signs of nearby alternate plants, colonizers will remain or repeatedly climb on an isolated plant accumulating the largest proportion of beetles retained on the release plant within a day. The data suggest that beetles that have climbed down the stem of the release plant will tend to climb back up the same plant unless nearby stems or canopy bridges are available.

The absence of a relationship between residency time and plant size even in the potato plot treatment supports the suggestion of Bach [15] that increased plant biomass does not result in longer CPB stay. Adult CPB have the ability to search a plant rapidly and actually spend most of their time feeding and resting on plants [16]. The outer boundary of the canopy on the single plant (L1) or on the three connected plants (L2) will determine the size of the potato unit searched but the largest impact on the residency period will come from other abiotic factors such as phototaxis [16] and biotic factors such as search for mates, need to oviposit and escape from predators. Regardless of treatment, the residency time of males was consistently shorter than that of females. This is consistent with the statement in the literature that males are more mobile than females in part as they search for mates [8].

In addition to the connectivity of the plants, CPB retention in the host patch would be enhanced by mixed borders acting as barriers to the dispersal of walking beetles to the outside of the patch. Mixed borders of all types tested (potato-woodland, potato-timothy and potato-bare ground) in our 2011 experiment were highly impermeable barriers to walking beetles suggesting that the insect responded to the discontinuity of the host canopy. The impermeability of mixed borders to walking beetles measured over a period of six hours in 2011 was maintained over periods of up to five days in 2012. This was in sharp contrast to the potato-potato borders which were highly permeable (Permeability index  = 0.95). The avoidance and detection of borders with very different vegetative or structural properties such as woodland habitat may not be entirely surprising but an intermediate number of crossings might have been expected into the less contrasting habitat of the timothy plots. Schmera [18] had observed significantly fewer movements of marked and released CPB between small groups of potato plants dispersed throughout a wheat field than between similar groups of potato plants in a bare ground matrix. These results provide background to their suggestion that beetles responded to the contrast between the matrix of wheat and fallow field. In arable fields without any nearby mixed - borders the frequency distribution of turning angles for colony, post-diapause, and summer CPB released at its center is uniform [43]. In contrast, in our host patches surrounded by mixed - borders, the frequency distribution of turning angles was clearly non-uniform. Our results show that the transition from the potato host crop habitat to bare ground or a non-host habitat acting as a barrier to emigration transformed the walking path of adult CPB. Radar observations of CPB walking tracks in the 2012 experiment revealed that the high frequency of moves in the direction of borders shifted to a dominance of moves away from or parallel to the mixed borders as they approached the edge of the patch. Walking adult CPB seemed to avoid the boundary between host and non-host habitat without having to cross it. As Conradt [44] noted for butterflies, the walking beetles seemed to control their rate of border crossing. Crossings into unsuitable habitat would be for dispersal rather than food plant search (see Conradt and Roper [44]).

The decreasing length of the perimeter relative to an increasing patch area combined with the strong barrier effect of the perimeter suggested that patch size would affect walking beetle retention. However, all 2012 field observations, except for Day 4 in July - August, indicated no effect of field size on beetle retention and that beetles dispersed away from the potato crop at the same rate regardless of patch size. The higher retention of beetles in small plots on day four in July - August corresponded to an overall lower retention of tagged beetles (Total 179) compared to the June - July (Total 335) and August (Total 283). The smaller sample size combined with more frequent rainfall during this period could have also contributed to the observed pattern.

The 2012 average retention rates of 37, 26 and 28% of released beetles after five days in small, medium and large plots, respectively, seemed relatively low considering the impermeability of the mixed-borders measured in 2011. Retention of tagged beetles in the plots was likely slightly higher than recorded taking into account the inevitable detection errors by the radar because of lost tags or a proportion of tagged beetles hidden in soil crevices or behind thick foliage during monitoring. Regardless, the departure rate is higher than expected considering the important role of crop borders in blocking emigration of walking beetles.

There are a few studies where adult CPB were released and emigration monitored from which retention rates can be extrapolated. For example, Williams [45] obtained a retention rate of 32% (disappearance rate of 68%) within the first 24 h after release in an eggplant plot. Follett [46] reported a minimum retention rate of 30% (maximum disappearance rate of 70%). Weber and Ferro [10] obtained retention rates of 23 and 46%, four days after release, in a fallow field and a potato field, respectively. Sandeson [47] obtained a retention rate of 5–30%, five days after release on potato. Szendrei [19] obtained retention rates near 40% for up to 178 h after release. The low retention rates obtained in our 2012 experiment are therefore realistic. Szendrei [19] did, however, note a significantly higher retention rate with recently emerged overwintered beetles than with older overwintered and summer generation beetles. The absence of a seasonal difference in our study could have resulted from the use of laboratory colony beetles throughout. The retention times observed by Szendrei [19] were similar whether potato were grown on bare ground, rye or vetch covered soil contrary to Schmera [18] who had reported higher retention rate in wheat covered ground than in fallow ground.

We suggest that the low beetle retention rate, in spite of the impermeability of mixed borders, resulted from a high departure rate by flight. The impermeability of mixed - borders, the fact that beetle retention was unaffected by a 16 fold difference in host patch size, the very low numbers of tagged beetles recovered from the trenches surrounding the potato patches in 2012 and the fact that none of the tracked beetles were observed walking across the mixed borders combine to suggest that much of the dispersal out of the plots occurred by flight rather than by walking. Many studies have reported on how unfavourable (hard) boundaries [26] between contrasting habitats limit the frequency of crossings for a range of insect species (see Skorva [7]) but our results highlight, perhaps for the first time, how these boundaries may impact differently walking and flying individuals of a same species.

The insect pest is more frequently sighted walking than flying because abiotic and biotic conditions favorable to adult CPB dispersal are generally less restrictive for walking than for flight [8]. For example, adult CPB will disperse by walking at temperatures as low as 15°C [48] but will rarely fly at temperatures below 24°C [49]. Also, the CPB is not a spontaneous flyer [50] and will drop and feign death rather than take flight to avoid predation or vibrations [51], [52]. Together these conditions may have led to an underestimation of the role of flight in CPB emigration from the host patch. Based on our results, factors such as plant connectivity and mixed border barriers may act together to reduce the level of emigration from the crop by walking beetle.

Also, the high rates of beetle departure observed consistently across patch sizes in spite of the small number of beetles walking across mixed borders suggest that mixed borders was not a barrier to CPB dispersal by flight. Although flight was visually observed on occasion throughout the 2011 and 2012 field experiments, it was not possible to systematically document it. Essentially, it would seem that the emigration rate of the beetle depended more on factors providing an incentive to leave than on the probability of encountering a mixed border.

### Implications for IPM

Our results may have an impact on how we model CPB dispersal throughout the landscape. It is frequently assumed in ecology that departure rates from habitat patches depend on the rate of chance encounters with habitat borders and the assumption then used to predict emigration rates based on circumference: area ratios of habitat patches [44]. As observed for two butterfly species in the prairies [44], awareness of host patch borders and lack of relationship between number of crossings and number of encounters with borders would preclude such assumption. Our results also imply that modification of the ratio of habitat edge to host patch area is not likely to be useful as a pest management tool. Results suggest that patch size manipulation could only play a minimal role in preventing further emigration from fields.

A model developed by Hannunen [53] suggested that changing the ratio of trap crop to crop area might improve the efficacy of the control method for some pests. Unfortunately, results of our study suggest that this is not likely to improve their efficacy in the case of the CPB. The impact of habitat manipulation (e.g. different distance between crop fields of different sizes) on the survival of the migrants remains to be evaluated and would seem a more promising avenue of study for long term beetle control.
